# Differential Mechanisms Drive Species Loss Under Artificial Shade and Fertilization in the Alpine Meadow of the Tibetan Plateau

**DOI:** 10.3389/fpls.2022.832473

**Published:** 2022-02-08

**Authors:** Junyong Li, Lachlan S. Charles, Zhongling Yang, Guozhen Du, Shenglei Fu

**Affiliations:** ^1^Dabieshan National Observation and Research Field Station of Forest Ecosystem, Henan University, Kaifeng, China; ^2^College of Geography and Environmental Science, Henan University, Kaifeng, China; ^3^School of Life Sciences, Henan University, Kaifeng, China; ^4^Terrestrial Ecosystem Research Network, Indooroopilly, QLD, Australia; ^5^School of Life Sciences, Lanzhou University, Lanzhou, China

**Keywords:** alpine meadow, fertilization, functional traits, artificial shade, species loss, resource competition

## Abstract

Fertilization is an effective management strategy to promote community biomass but can simultaneously reduce species diversity in many grassland systems. Shifts in competition for resources have been proposed to explain the decline in plant species diversity due to fertilization, yet the underlying mechanism driving species loss remains controversial. This uncertainty may be driven by variation in aboveground and belowground resource availability. However, experiments simultaneously manipulating both light availability and soil nutrients are rare. Using a 6-year field experiment to manipulate light availability (*via* shade cloth) and soil nutrients (*via* fertilizer addition), we tested this resource competition hypothesis in a species-rich alpine meadow by examining the variation of species traits associated with the capacity of light acquisition within these treatments. Our results showed that artificial shade decreased community biomass accumulation whereas fertilization increased it. In contrast, both shade and fertilization reduced species diversity. Extinction of non-*Gramineae* species (e.g., *Fabaceae* and *Cyperaceae*) was the main reason for species diversity decline. Species loss can be explained by the limitation of light availability and predicted by species traits associated with light acquisition capability under fertilization and low light tolerance under artificial shade. Specifically, fertilization eliminated species with lower stature and artificial shade exterminated species with the higher light compensation point (LCP). The findings suggest that light availability is consistently important for plant growth and that low competitiveness for light under fertilization and intolerance of low light conditions under artificial shade trigger species loss process in the alpine meadow. Our experiment helps clarify the mechanisms of how artificial shade and fertilization decreased species diversity and highlight that LCP, which tends to be neglected by most of the studies, is one of the vital drivers in determining species coexistence.

## Introduction

Human activities, such as industrial agriculture, are altering terrestrial nutrient availability worldwide by widespread fertilizer application (Yang et al., 2013; IPCC, 2014; FAO et al., 2017). Fertilizer application is a common and efficient management practice to promote productivity but reduces plant species diversity in grassland ecosystems. However, understanding the specific mechanisms of species loss in response to nutrient enrichment remains a challenge for ecologists (Isbell et al., 2013; Harpole et al., 2016; DeMalach et al., 2017).

The decline of plant species diversity at high soil fertility or high productivity is thought to be a result of increased competition for light (Hautier et al., 2009; DeMalach et al., 2017). The light competition hypothesis predicts that competition may shift from belowground when soil resources are limited to aboveground when soil resources are abundant but shading is intense (Newman, 1973). This reduction in photosynthetically active radiation (PAR) may, over time, lead to the competitive exclusion of shade-intolerant species and improved fitness for light resource acquisitive species (Tilman, 1982; Hautier et al., 2009).

While previous studies have observed species decline due to a reduction in light availability after fertilizer application (Carson and Pickett, 1990; Hautier et al., 2009), there is strong evidence that an increase in nutrient availability after fertilization decreased plant species diversity even when the light was not limiting (Harpole and Tilman, 2007; Dickson and Foster, 2011; Borer et al., 2014; Yang et al., 2015; Harpole et al., 2016). This pattern was further supported by two short-term (≤2 years) field experiments, which used shade cloth to reduce light availability without changing species diversity (Rajaniemi, 2002; Li et al., 2011).

While the majority of experimental approaches used to investigate the relationship between light competition and diversity loss are derived from community-level studies (Harpole et al., 2016; DeMalach et al., 2017; Li et al., 2017), fewer studies have focused on individual-level responses to nutrient enrichment and light limitation based on functional traits associated with light acquisition (Suding et al., 2005; Dickson et al., 2014; Yang et al., 2015). For example, plant height is a well-documented trait that relates to improve light acquisition (Grime, 2001), with taller plants having an advantage in low-light conditions driven by the increase of community biomass in response to fertilization (Hautier et al., 2009; Borer et al., 2014). Another key functional trait affecting the survival of plant species under light-limiting conditions is light compensation point (LCP), which is defined as the minimum light level required for plant survival and regular growth. LCP reflects the shade tolerance of a species (Horn, 1971), and species with low LCP typically correspond to an increase in carbon acquisition, higher shade tolerance, and high survival probability under limited light availability conditions (Kitao et al., 2016). Thus, functional traits associated with light capture and shade tolerance may dictate species performance in response to reduced light availability.

Alpine meadows within the Tibetan Plateau are a climate-sensitive ecosystem and currently face the selection pressures of increasing nutrient loading (IPCC, 2014; Zhang et al., 2019; Ma et al., 2021; Sun et al., 2021a,b). Understanding the mechanism controlling species diversity is essential for maintaining ecological equilibrium in this sensitive ecosystem. However, the underlying mechanisms driving species loss after nutrient addition remain controversial. In this study, we presented results of the field experiment of 6 consecutive years by manipulating light availability indirectly *via* increasing soil resource availability and directly *via* shade cloth in an alpine meadow of the Tibetan Plateau. We compared the effects of nutrient addition and artificial shade on species diversity and community composition and then, explored the relationships between species relative abundance (SRA) and functional traits to address the following three questions. (1) How does plant species diversity respond to changes in light and nutrient availability? (2) Do changes in community composition within the artificial shade and fertilization treatment convergent or divergent along a temporal scale? and (3) Are the functional traits associated with light acquisition capability and the minimum light level required for photosynthesis good indicators to predict species loss in the alpine meadow of the Tibetan Plateau?

## Materials and Methods

### Study Site

This experiment was performed at the Research Station of Alpine Meadow and Wetland Ecosystems of Lanzhou University located in Maqu County (34°00′N, 102°00′E; 3,500 m above the sea level), eastern Tibetan Plateau, China. The annual average temperature during the sampling period (2008–2012) ranged from −8.2°C in winter to 11.7°C in summer, with the annual average precipitation of 706 mm, mainly distributed during the short, cool summer (Supplementary Figure 1). The vegetation, typical alpine meadows of the Tibetan Plateau, is dominated by perennial sedges (e.g., *Kobresia graminifolia*), grasses (e.g., *Poa botryoides and Elymus nutans*), species of *Compositae* (e.g., *Saussurea nigrescens*), and other broad-leaved species (e.g., *Anemone rivularis*). The average aboveground biomass within the experimental site (450 m × 220 m) ranges from 280 to 400 g m^–2^ dry weight, which corresponds to the median value of global grassland productivity; and the species richness ranges from 20 to 35 per 0.25 m^2^, at the upper limit of species diversity among the global grassland ecosystems (Borer et al., 2014).

### Experimental Design

To assess the impact of light and soil nutrient availability on species diversity, two blocks (25 m × 30 m) were established within the fenced exclosure (450 m × 220 m) to exclude herbivores. Light availability was manipulated by covering one block with a black polypropylene mesh shade cloth, which was permeable to air and water, to reduce 70 ± 2% (mean ± SD) of PAR. The shade cloth was suspended 1.3 m above the ground surface by wooden stakes attached to the corners of one block and fastened to the ground on all sides. The other block remained uncovered, corresponding to ambient light availability. Nutrient availability was manipulated by two fertilizer addition levels: 0 g m^–2^ year^–1^ (no fertilizer addition) and 45 g m^–2^ year^–1^ of a slow-release pelletized fertilizer [(NH_4_)_2_HPO_4_] manufactured by Tianjin International Trading Company, Tianjin, China, corresponding to 9.5 g N m^–2^ and 10.6 g P m^–2^. The nutrient treatment was nested within the light availability treatment block, resulting in a total of four treatments: fertilizer addition and ambient light (F); no fertilizer addition and shade (S); synchronous fertilizer addition and shade (F+S); and no fertilizer addition and ambient light (CK). Thirty-two permanent 2 × 2 m plots were established with 16 plots under the shade shed and the other 16 plots outside the shed, arranged in a regular 4 × 4 matrix with a 2-m buffer zone between plots. Half of the plots both inside and outside of the shade shed were randomly selected to add fertilizer, which means that each treatment had eight replications (refer to Supplementary Figure 2, for more details). The experiment began with a background survey (*n* = 12) to evaluate the entire experimental community characteristics of the location site on August 25, 2007. Shade and fertilization treatments were applied annually at the beginning of each growing season (end of May) from 2008 to 2012. Artificial shade shed was dismissed annually during the nongrowing season (usually from October to next May) to avoid destruction by local strong wind and heavy snow.

### Species Diversity and Aboveground Biomass

To measure species diversity, one quadrat (0.5 m × 0.5 m) was randomly selected in each plot at the end of each growing season (September), approximately 0.5 m away from the edge to avoid the edge effect. Within each quadrat, species were identified, and the number of all individuals was recorded. For clonal species, we regarded a ramet as an individual (Yang et al., 2013). To quantify aboveground biomass, the aboveground parts of all plant individuals rooted within each quadrat were harvested. All samples were dried at 60°C for 72 h and weighed (accuracy of 10^–2^ g). We separated all species into two functional groups: *Gramineae* and non-*Gramineae* groups, which generally correspond with their appearance in the canopy and understory within the quadrats, respectively. Within the non-*Gramineae* group, species belonging to *Cyperaceae* and *Fabaceae* families were analyzed as separate subgroups since these species are known to be sensitive to light resource availability (Supplementary Table 1; Li et al., 2011).

### Belowground Biomass and Soil Nutrients

To assess belowground biomass and soil nutrients, we employed the root in-growth method (3.5 cm diameter × 20 cm deep) to estimate the belowground biomass from each quadrat post aboveground harvesting in 2012. Soil samples were brought to the laboratory in air-tight plastic bags, and then, roots were washed from the soil cores with a 2-mm mesh sieve after air-drying. Root samples were dried at 60°C for 72 h and weighed (accuracy of 10^–2^ g). Soil characteristics (including pH, total nitrogen, total phosphorus, total organic carbon, available nitrogen, and available phosphorus) were analyzed using the protocol described by Liu et al. (2015).

### Light Availability

Light availability was measured in each plot using a Decagon Sunfleck Ceptometer (Decagon, Pullman, Washington, DC, United States) in August 2012. Light readings were taken on a cloudless day between 11:00 and 13:00 h. The ceptometer was placed north-south across each plot, and PAR was recorded at 0, 10, and 40 cm above the soil surface (below vegetation canopy). Additionally, PAR above the vegetation canopy was also recorded in each plot as a measure of full sunlight. The soil temperature was not monitored during the experimental period for the reason that the shade shed was permeable to air and water and suspended 1.3 m above the ground, resulting in no significant difference with fertilization plots in soil moisture and understory PAR (Table 1).

**TABLE 1:** Light intensity and soil property in different treatments and the results of two-way ANOVA for the effects of fertilization (F), shade (S), and their interaction.

Environmental variables									
	PAR (0 cm HAG) (μmol m^–2^ s^–1^)	PAR (10 cm HAG) (μmol m^–2^ s^–1^)	PAR (40 cm HAG) (μmol m^–2^ s^–1^)	Light availability score	Moisture (%)	pH	SOC (%)	SAN (mg kg^–1^)	SAP (mg kg^–1^)
Control (CK)	410.38 ± 34.72a	1212.13 ± 85.64a	5965.50 ± 102.15a	1.64 ± 0.13a	42.2 ± 1.45a	7.12 ± 0.02a	2.05 ± 0.11a	14.45 ± 0.19c	1.76 ± 0.07b
Fertilization (F)	107.13 ± 5.60b	332.00 ± 65.69b	3248.25 ± 112.89b	−0.24 ± 0.05b	42.4 ± 0.99a	6.91 ± 0.17a	1.97 ± 0.12a	17.94 ± 0.59b	63.68 ± 6.09a
Shade (S)	117.25 ± 7.57b	238.00 ± 16.32b	1441.50 ± 17.06c	−0.62 ± 0.02c	40.4 ± 2.92a	7.09 ± 0.12a	1.85 ± 0.04a	14.65 ± 0.24c	1.82 ± 0.24b
F+S	43.00 ± 4.30c	238.75 ± 14.83b	1472.25 ± 134.42c	−0.78 ± 0.03c	44.5 ± 1.33a	6.96 ± 0.07a	1.95 ± 0.08a	23.47 ± 1.07a	64.81 ± 1.65a
**Summary of two-way ANOVA**									
Effect of F	***F* = 108.54 *P* < 0.001**	***F* = 63.72 *P* < 0.001**	***F* = 173.76 *P* < 0.001**	***F* = 205.95 *P* < 0.001**	*F* = 1.38 *P* = 0.258	*F* = 2.58 *P* = 0.128	*F* = 0.03 *P* = 0.878	***F* = 81.96 *P* < 0.001**	***F* = 493.64 *P* < 0.001**
Effect of S	***F* = 97.21 *P* < 0.001**	***F* = 93.88 *P* < 0.001**	***F* = 955.53 *P* < 0.001**	***F* = 387.89 *P* < 0.001**	*F* = 0.01 *P* = 0.931	*F* = 0.01 *P* = 0.949	*F* = 1.40 *P* = 0.255	*F* = 1.77 *P* = 0.284	*F* = 0.05 *P* = 0.830
Effect of F × S	***F* = 39.94 *P* < 0.001**	***F* = 63.94 *P* < 0.001**	***F* = 181.80 *P* < 0.001**	***F* = 145.36 *P* < 0.001**	*F* = 1.13 *P* = 0.305	*F* = 0.13 *P* = 0.722	*F* = 1.01 *P* = 0.329	***F* = 15.37 *P* = 0.001**	*F* = 0.04 *P* = 0.850

*Data were means ± SE (n = 8) collected from the last year of the experiment (2012). PAR, HAG, SOC, SAN, and SAP stand for photosynthetically active radiation, height of above the ground, soil organic carbon, soil available nitrogen, and soil available phosphorus, respectively. Significant differences among treatments within each variable were determined using the least-significant difference (LSD) test (P ≤ 0.05) and indicated by different letters. Significant effects are in bold. PCA ordinations showed that the variations of light (83.0%) could be well explained by the light availability score (PC1).*

### Trait Measurements

Prior to the community biomass collection in 2012, we selected 14 common species in fertilization plots, 17 species in shade plots, 7 species in synchronous shade and fertilization plots, and 21 species in control plots to measure functional traits (Supplementary Table 1). These species accounted for more than 92.4, 89.2, 94.7, and 82.5% of the aboveground biomass in fertilized, shaded, simultaneous shade and fertilization and the control communities, respectively. For each treatment, we measured the maximum height of 24 individuals (3 individuals per plot) of each species. The photosynthesis-light response curve of each species was measured for 3 fully expanded leaves using the LICO LI-6400 portable photosynthesis system. PAR levels of 0, 20, 50, 80, 100, 150, 200, 400, 800, 1,600, and 1,800 μmol m^–2^ s^–1^ were provided by red light-emitting diodes. Ambient air in the leaf chamber was maintained at 20°C, and the CO_2_ concentration of the incoming air was controlled at 400 μmol L^–1^. Parameters of photosynthesis-light response curve were simulated by the nonrectangular hyperbola equation using SPSS software (version 22.0). The model is given as follows:


$$A=\left(a\,P\,A\,R+A\,m\,a\,x-\sqrt{\left({\left(a\,P\,A\,R+A\,m\,a\,x\right)}^{2}-4\,k\,a\,P\,A\,R\times A\,m\,a\,x\right)}\right)/2\,k-R\,d\,a\,y$$

where *A* is the net leaf photosynthesis rate (μmol m^–2^ s^–1^), Amax is the light-saturated net leaf photosynthesis rate (μmol m^–2^ s^–1^), *a* is the light-limited quantum efficiency (μmol CO_2_ μmol^–1^ photons), *k* is the curvature parameter, and Rday is the dark respiration rate. When *A* is zero, the PAR is the LCP.

### Data Analysis

Two plant diversity indexes are calculated to describe the species diversity within each treatment plot. One is species richness, which is defined as the number of species present in a quadrat and the other one is the Shannon–Weiner index (*H*), which is calculated as:


$$\text{H}=-\sum_{i-1}^{s}\left(p_{i}\right)\,\left(\ln p_{i}\right)$$

where *p*_*i*_ is the relative abundance of species *i* in a quadrat and *s* is the species richness in the quadrat. We also calculated SRA (SRA = number of individuals of a given species in a quadrat/total number of individuals for all species in the quadrat) for each treatment plot.

Prior to analysis, all response variables were tested to meet the assumptions of normality and heterogeneity of variance. Any variable not meeting these assumptions was log-transformed. To account for differences in light resource availability at different levels of the canopy, the scores of the first principle component (PC1) of principal component analysis (PCA) were viewed as a proxy of light availability (Liu et al., 2015). The PC1 described 83% of the variation in light availability at different heights above the soil surface (Supplementary Figure 3). First, two-way ANOVA was performed to test the effects of fertilization and shade on belowground biomass and local abiotic conditions because all the above response variables were only measured in the last year of the experiment (2012). Second, we used repeated measure ANOVA to test for the significance of the effects of fertilization and shade on plant community characteristics across the 5 years (2008–2012). The years were used as within-subject factors, and fertilization and shade were used as between-subject factors. Third, the significant differences of each response variables among treatment plots were determined using the least significant difference (LSD) test at the 95% CI. Linear regression was used to assess the relationship between light availability and species diversity and plant functional traits and SRA. Fourth, we used paired-samples *t*-test (2-tailed) to test for differences of plant functional traits of *Gramineae* and non-*Gramineae* groups among the treatment plots at the community level. All the above statistical analyses were performed using SPSS version 22.0 (SPSS Inc., Chicago, IL, United States).

To assess the differences in community composition between the treatment plots, we computed the dissimilarities of plant species composition among different treatment plots along temporal scale by nonmetric multidimensional scaling (NMDS) with Bray-Curtis distance using the “metaMDS” function of the R package “Vegan” (Oksanen et al., 2010). The statistical method can depict community composition in multidimensions, and the variance of samples is maximized on the first dimension (Oksanen et al., 2010). Ordination was constructed in two dimensions and calculated with R-program using Windows version 3.4.2.

Structural equation modeling (SEM) was employed to examine the relationships of fertilization, shade, and species functional traits and SRA among *Gramineae* and non-*Gramineae* groups, respectively. The pathways through which fertilization and shade influence SRA were also assessed (Shipley, 2002). Two categorical variables were chosen to describe the treatments: fertilization (0 = no fertilizer addition; 1 = fertilization) and shade (0 = no shade cloth; 1 = shade). Results from the best fitness SEM showed that fertilization and shade affect SRA by changing species height and LCP (indicated by one-way arrows in the path diagrams). The method has been widely used previously (Socher et al., 2012; Li et al., 2017). All SEMs were performed using AMOS 20.0 (SPSS Inc., Chicago, IL, United States).

## Results

### Effects of Fertilization and Shade on Local Abiotic Variables

The light intensity varied among 0, 10, and 40 cm layers of the community in the control plots (*F* = 581.3, *P* < 0.001) and were significantly affected by fertilization, shade, and their interactions (all *P* < 0.01). On average, light intensities (0–40 cm heights above the ground) were 2,529 ± 74 μmol m^–2^ s^–1^ in the control plots. Fertilization, shade, and F+S treatments significantly reduced light intensity by 51.4, 76.3, and 76.9%, respectively (all *P* < 0.001, Table 1). Within the control plots, soil available nitrogen (SAN) and soil available phosphorous (SAP) were recorded as 14.45 ± 0.19 and 1.76 ± 0.07 mg kg^–1^, respectively. Fertilization dramatically increased SAN and SAP by 24.2 and 3,518.2% (both *P* < 0.001), respectively. In addition, shade treatment had no significant effects on either SAN or SAP (both *P* > 0.05). There was a significant interactive effect of shade and fertilization on SAN (*P* = 0.001), yet the interaction effect was not detected on SAP (*P* > 0.05). Neither fertilization nor shade treatment significantly affected soil moisture, soil pH, and soil organic carbon (all *P* > 0.05, Table 1).

### Effects of Fertilization and Shade on Species Diversity and Community Variables

#### Species Diversity

**FIGURE 1:**
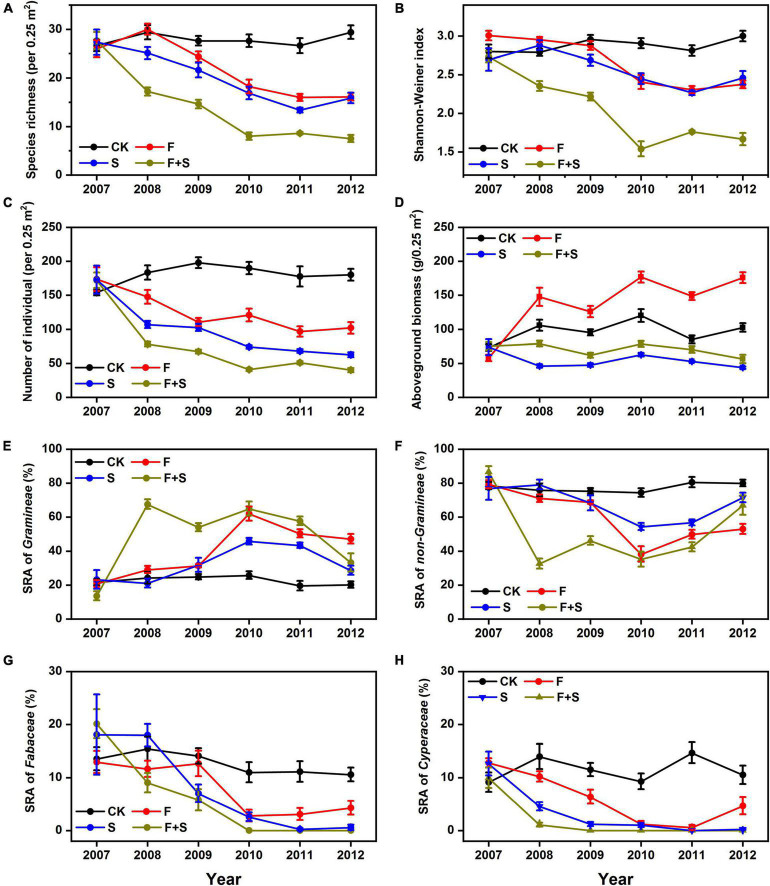
The effect of fertilization (F, red line), shade (S, blue line), and interaction of fertilization and shade (F+S, brown line) on temporal trends of species richness **(A)**, Shannon–Weiner index **(B)**, number of individuals **(C)**, aboveground biomass **(D)**, species relative abundance (SRA) of *Gramineae* **(E)**, SRA of non-*Gramineae* **(F)**, SRA of *Fabaceae* **(G)**, and SRA of *Cyperaceae* **(H)**. Circular symbols represent means and SE (*n* = 8) across 6 years (2007–2012). The Control treatment (CK = ambient light and no fertilizer addition) is denoted by black lines. Means were connected by lines to illustrate the temporal patterns. Means and SE of 2007 were calculated using the baseline surveys prior to treatments (*n* = 3) (refer to the section “Materials and Methods” for more details).

A total of 40 species belonging to 18 families were recorded, and no significant effect of year on species richness and Shannon–Weiner index was detected in the control plots across the 6 years (both *P* > 0.05, Supplementary Table 1). In contrast, species richness and Shannon–Weiner index were reduced by an average of 26.0 and 10.7% in fertilization plots, 33.9 and 11.7% in shade plots, 61.1 and 34.6% in F+S plots, respectively (Figures 1A,B and Table 2). The number of individuals per quadrat was also significantly reduced by an average of 33.4% in fertilization plots, 57.9% in shade plots, 72.1% in F+S plots, respectively (Figure 1C and Table 2).

**TABLE 2:** Plant community characteristics (mean ± SE) in different treatments and the results of repeated measure ANOVA (*F*-value and degrees of freedom) for the effects of the fertilization and shade on plant community characteristics.

Plant variables	Community characteristics in different treatments				Summary of the results of repeated measure ANOVA						
		
	Control	Fertilization (F)	Shade (S)	F+S	Year (Y)	Fertilization (F)	Shade (S)	Y × F	Y × S	F × S	Y × F × S
											
*d.f.*					4	1	1	4	4	1	4
Species richness	28.35 ± 0.61a	20.98 ± 1.00b	18.75 ± 0.88b	11.03 ± 0.67c	53.27***	161.32***	270.42***	7.22***	2.06^ns^	0.09^ns^	10.60***
Shannon–Weiner index	2.89 ± 0.03a	2.58 ± 0.05b	2.55 ± 0.05b	1.89 ± 0.06c	49.48***	172.13***	189.92***	14.65***	7.19***	21.38***	6.20***
Number of individual	199.0 ± 5.9a	132.5 ± 8.9b	83.7 ± 3.6c	55.5 ± 2.8d	76.31***	114.21***	470.80***	5.28**	19.08***	18.71***	7.17***
Aboveground biomass	100.7 ± 3.6b	154.1 ± 5.3a	50.1 ± 1.4d	69.3 ± 2.5c	7.39***	202.37***	705.82***	2.87*	5.35**	44.72***	3.45*
Belowground biomass	736.1 ± 50.7a	745.1 ± 36.3a	414.8 ± 23.0b	332.1 ± 25.0b	–	1.08^ns^	106.95***	–	–	1.67^ns^	–
Root:Shoot ratio	7.71 ± 0.60a	4.32 ± 0.34b	8.00 ± 1.04a	7.66 ± 0.63a	–	7.12*	6.75*	–	–	4.76*	–
SRA (*Gramineae*)	22.82 ± 1.20d	43.90 ± 2.32b	34.05 ± 1.93c	55.41 ± 2.54a	21.34***	190.01***	54.56***	3.82**	7.32***	0.01^ns^	20.68***
SRA (Non-*Gramineae*)	77.18 ± 1.20a	56.10 ± 2.32c	65.95 ± 1.93b	44.59 ± 2.54d	21.34***	190.02***	54.56***	3.82**	7.32***	0.01^ns^	20.68***
SRA (*Fabaceae*)	11.96 ± 0.88a	4.58 ± 0.75b	1.34 ± 0.33c	0.21 ± 0.10c	6.45**	50.23***	155.50***	2.08^ns^	2.42^ns^	50.17***	3.94*
SRA (*Cyperaceae*)	12.41 ± 0.88a	6.86 ± 0.99b	5.65 ± 1.21bc	2.95 ± 0.82c	27.83***	21.71***	36.41***	1.40^ns^	3.66*	38.42***	2.96*

Community characteristics were collected and calculated with the data (n = 40) across 5 experimental years (2008–2012) except for that belowground biomass and root:shoot ratio were collected and calculated using the data (n = 8) from the last year of the experiment (2012). Significant differences of each community characteristic among treatments were determined using the least-significant difference (LSD) test (P ≤ 0.05) and indicated by different letters. The value of species richness and number of individuals are numbers per 0.25 m^2^ (a quadrat area), and biomass is denoted in grams per 0.25 m^2^. *P ≤ 0.05, **P ≤ 0.01, ***P ≤ 0.001; ns, not significant. SRA, species relative abundance; d.f., degree of freedom.

#### Community Biomass

Fertilization significantly increased aboveground biomass by 53.0% but had no significant effect on belowground biomass, resulting in a 44.0% decrease in the root:shoot ratio, while shade significantly decreased aboveground by 50.2% and belowground biomass by 43.6%, resulting in a neutral influence on the root:shoot ratio. The effect F+S treatment significantly decreased aboveground by 31.2% and belowground biomass by 54.9%, resulting in a neutral influence on the root:shoot ratio as same as the shade treatment (Figure 1D and Table 2).

#### Species Relative Abundance

Compared with the control plots, the SRA of *Gramineae* showed a significant increase in response to fertilization (92.4%), shade (49.2%), and F+S treatment (142.8%), and the SRA of non-*Gramineae* inevitably decreased in response to fertilization (27.3%), shade (14.6%), and F+S treatment (42.2%) (Figures 1E,F and Table 2). The treatments performed a remarkable influence on the SRA of the two groups along the temporal scale, especially for the *Fabaceae* and *Cyperaceae* within the non-*Gramineae* group, (Figures 1G,H).

#### Community Composition

**FIGURE 2:**
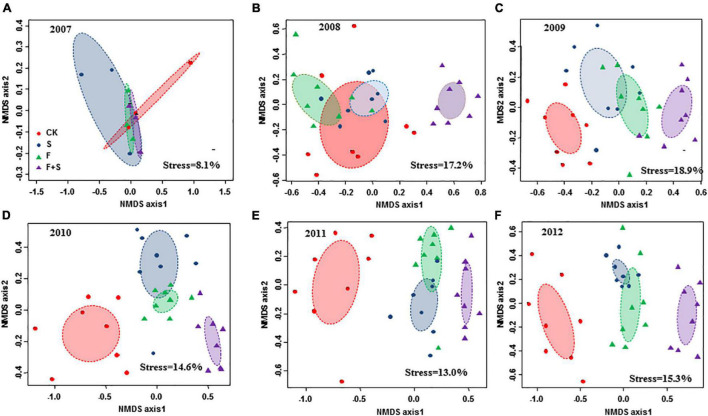
Nonmetric multidimensional scaling (NMDS) patterns of community dissimilarities among treatments using each quadrat data (*n* = 8) of plant species composition along the temporal scale (**A**: 2007; **B**: 2008; **C**: 2009; **D**: 2010; **E**: 2011; **F**: 2012). Ellipses with different colors indicate 95% CI ellipses for centroids of the control (CK, red color), fertilization (F, green color), shade (S, blue color), and interaction with fertilization and shade (F+S, purple color). It is noted that quadrat data of 2007 (*n* = 3) were calculated using the baseline surveys prior to treatments (refer to the section “Materials and Methods” for more details).

Nonmetric multidimensional scaling ordinations of plant communities revealed that prior to the implementation of treatments, there was no distinct separation of species composition among the initial communities (Figure 2A). Since 2008, significant separation trends among different treatment plots were gradually exhibited along the first two dimensions of the NMDS over time (Figures 2B–F). Plant community similarity in all treatment plots diverged from control plots, whereas composition did not significantly differ between fertilization plots and shade plots. Dominant species shifted from a mixture of *Trigonella ruthenica (Fabaceae), Kobresia capillifolia (Cyperaceae), and E. nutans (Gramineae)* in control plots to *Gramineae* species with high height (*Poa poophagorum* and *Koeleria cristata*) and non-*Gramineae* shade-tolerant species (*Anemone obtusiloba*) in fertilization plots and shade plots (Supplementary Table 2).

### Effects of Fertilization and Shade on Species Functional Traits

Results of paired-samples *t*-test showed that shade significantly increased species height of *Gramineae* and non-*Gramineae*, while fertilization significantly increased species height of *Gramineae* but had no significant effect on non-*Gramineae* (Table 3 and Supplementary Table 2). Meanwhile, fertilization significantly increased the LCP of *Gramineae* and non-*Gramineae* groups, while shade significantly decreased LCP of non-*Gramineae* but had no significant effect on the *Gramineae* group. Neither species height nor LCP was significantly affected by the F+S treatment (Table 3 and Supplementary Table 2).

**TABLE 3:** Results of paired-samples *t*-test (2-tailed) for the effects of treatments on species height and light compensation point (LCP) of *Gramineae* and non-*Gramineae* species at the community level.

	*Gramineae*					Non-*Gramineae*				
Treatment	*d.f.*	Species height		LCP		*d.f.*	Species height		LCP	
		*t*	*p*	*t*	*p*		*t*	*p*	*t*	*p*
Fertilization (F)	4	**−5.651**	**0.005**	**−3.510**	**0.025**	8	−0.798	0.448	**−3.281**	**0.011**
Shade (S)	3	**−5.011**	**0.015**	2.736	0.072	12	**−5.210**	**0.001**	**5.502**	**0.001**
F+S	2	−3.332	0.079	2.205	0.158	3	−2.324	0.103	1.718	0.184

*Significant differences are denoted in bold (P < 0.05).*

### Relationships Among Abiotic Variables, Community Variables, and Functional Traits

**FIGURE 3:**
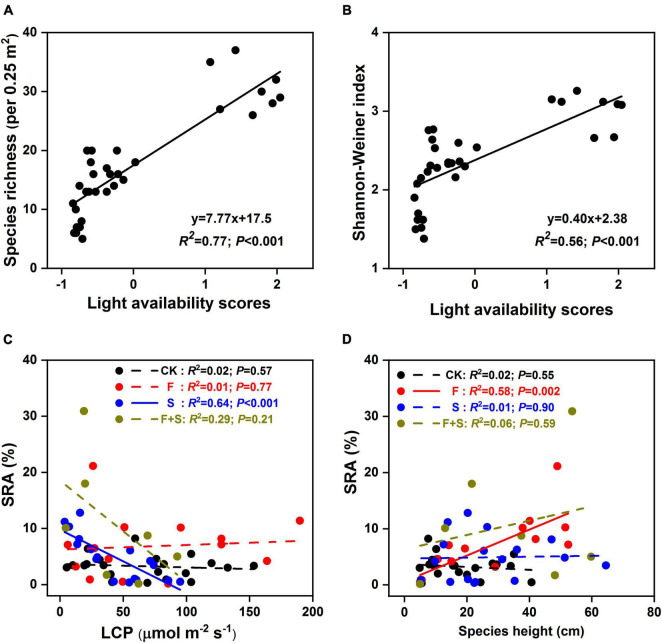
Linear regressions of light availability scores vs. species richness **(A)**, Shannon–Weiner index **(B)** and light compensation point (LCP) **(C)**, species height **(D)** vs. SRA among the control (CK, black), fertilization (F, red), shade (S, blue), and synchronous fertilization and shade (F+S, brown) plots, respectively. Symbols were means of the SRA of 21 species in control plots, 17 species in shade plots, 14 species in fertilized plots, and 7 species in F+S plots for panels **(C,D)**, respectively. Solid and dashed arrows indicate significant (*P* < 0.05) and nonsignificant relationships (*P* > 0.05) between the variables (refer to the section “Materials and Methods” for more detail).

Significantly positive correlations were detected between scores of light availability and species richness (*R*^2^ = 0.76, *P* < 0.001, Figure 3A) and Shannon–Weiner index (*R*^2^ = 0.55, *P* < 0.001, Figure 3B). There was a strong negative relationship between LCP and SRA in shade plots (*R*^2^ = 0.64, *P* < 0.001), but the relationship was not detected in the control, fertilization, or F+S plots (Figure 3C). Species height was positively correlated with SRA in fertilization plots (*R*^2^ = 0.58, *P* < 0.001), whereas no such relationship was observed in the control, shade, or F+S treatment plots (Figure 3D).

**FIGURE 4:**
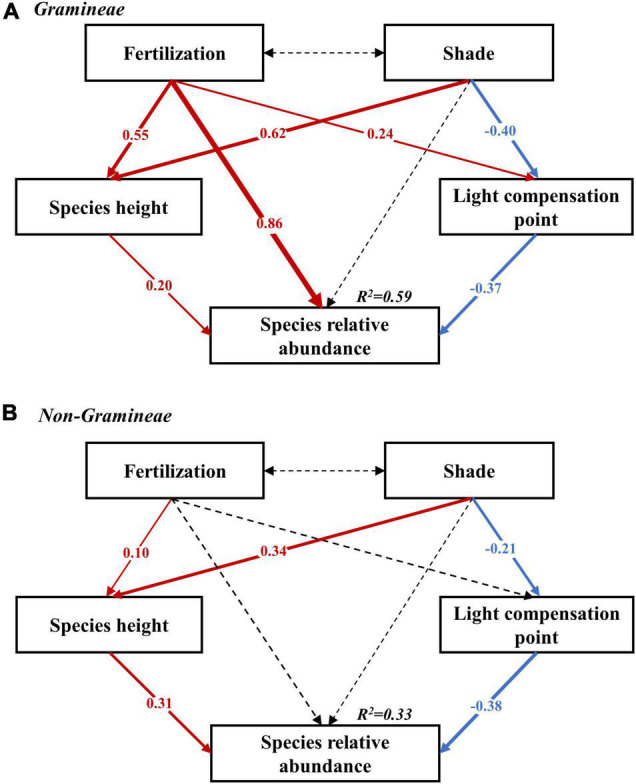
The results of the best-fitting structural equation model (SEM) showing the causal relationships among fertilization, shade, species height, LCP, and species relative abundance of *Gramineae* species and non-*Gramineae* species. **(A)** *Gramineae* species and model fit statistics variables were χ^2^ = 0.179, *P* = 0.672, CFI = 1.000, root mean square error of approximation (RMSEA) = 0.000, Akaike information criterion (AIC) = 38.179. **(B)** Non-*gramineae* species and model fit statistics variables were χ^2^ = 1.900, *P* = 0.167, CFI = 0.935, RMSEA = 0.151, AIC = 39.907. Solid and dashed arrows indicate significant (*P* < 0.05) and nonsignificant relationships (*P* > 0.05) between the variable of the onset arrow and the variable of the terminated arrow, respectively. The thickness of the arrows reflects the degree of relationships, and red and blue arrows indicate positive and negative relationships, respectively. Numbers at arrows are standardized path coefficients. *R*^2^ values indicate the variation of response variables explained by the models.

We employed SEM to further estimate the contribution of four factors (i.e., fertilization, shade, species height, and LCP) on the variation of SRA and found that the optimal SEM model explained 59% of the variations in SRA of *Gramineae* and 33% of the variations in SRA of non-*Gramineae* (Figure 4). For *Gramineae* species, fertilization had significant direct and indirect effects on SRA which was mediated by increasing species height and LCP, whereas shade treatment had significant indirect effects on SRA *via* increasing species height and decreasing LCP (Figure 4A). For non-*Gramineae*, fertilizer application had no significant direct effects but displayed significant indirect effects on SRA *via* increasing species height, whereas shade treatment had significant indirect effects on SRA *via* increasing species height and decreasing LCP (Figure 4B).

## Discussion

Overall, our 6-year field experiment *via* simultaneously manipulating the light availability and soil nutrient conditions revealed that artificial shade could reduce species diversity as same as fertilization in the alpine meadow. Reduction in light availability triggered species loss process both in the artificial shade and fertilization plots but through different mechanisms. Specifically, fertilization facilitated the survival of species with tall stature, whereas artificial shade promoted the performance of species with low LCP. Our results confirmed that species height and LCP were effective indicators of species coexistence patterns in the light limitation habitat of the Tibetan Plateau.

### Effects of Fertilization and Shade on Local Abiotic Conditions and Plant Diversity

The availability of light in the lower canopy layers influences conditions for species recruitment, growth, and reproduction, thus affecting plant diversity (Kotowski and van Diggelen, 2004). In our study, light intensity was significantly reduced directly by artificial shade and indirectly by fertilization *via* increasing aboveground biomass (Figure 1D and Table 1). As expected, artificial shade treatment had no influence on the abiotic conditions except for the light availability. In addition, fertilization significantly increased soil available nitrogen and soil available phosphorus (Table 1). These responses of environmental variables to experimental treatments verified our hypothesis assumption that fertilization decreased light availability in plant community and increased soil nutrient contents, while shade decreased light availability without changing soil nutrient contents (Table 1). Our results were consistent with the prediction of the light competition hypothesis (Rajaniemi, 2002), both fertilization and shade treatment significantly reduced species diversity (Figures 1A,B and Table 2), and species diversity was strongly positively correlated with light availability scores (Figures 3A,B), indicating that direct and indirect light limitation performed similarly negative effects on plant diversity regardless of aboveground biomass increased by fertilization or decreased by shade treatment (Figure 1D).

Interestingly, changes in species diversity due to reductions in light intensity were only apparent after the 2nd year of exposure to artificial shade or fertilization and became more pronounced along the temporal scale (Figures 1A,B). The result was consistent with previous studies that short-term (1–2 years) artificial shade had a neutral effect on species diversity (Rajaniemi, 2002; Li et al., 2011). Tilman (1988) reported that short-term community dynamics following disturbance was merely an intermediate process during the succession. Moreover, another research proved that it took over 10 years for the shifts in species composition occurring after fertilization in northwestern Canada (Turkington et al., 2010).

Our results also mirrored the commonly observed negative relationship between plant diversity and nutrient availability (Rajaniemi et al., 2003; Suding et al., 2005; Harpole and Tilman, 2007; Bobbink et al., 2010; Duprè et al., 2010; Isbell et al., 2013, 2015). In this study, an increase in nutrient availability and subsequent enhancement for aboveground biomass may increase competition intensity for light (Hautier et al., 2009; Borer et al., 2014; Yang et al., 2015), conferring a competitive advantage to fast-growing resource acquisitive species at the expense of slower growing, resource conservative species (Dybzinski and Tilman, 2007; Hautier et al., 2009; Dickson and Foster, 2011; DeMalach et al., 2017). It was noteworthy that the soil pH was not affected by fertilization in our experiment, contrary to other study reports (Clark et al., 2010; Li et al., 2011, 2017; Yang et al., 2021; Table 1). One possible reason was that we employed ammonium phosphate [(NH_4_)_2_HPO_4_], a slow-release pelletized fertilizer, to manipulate the soil nutrient input. The fertilizer is widely used in the local agricultural cultivation and showed weak alkalinity, while other studies involving fertilization usually employed ammonium nitrate (NH_4_NO_3_), strong acid and weak base salt, as fertilizer and showed that fertilization induced soil acidification and reduced species diversity, which differed with our experimental design. Overall, to the best of our knowledge, the result that artificial shade performed the same negative effect on species diversity as fertilization had not been reported in previous studies. Our results provide novel evidence that artificial shade had the same negative effect on species diversity as increased nutrient availability and that species loss can be simply triggered by light limitation, independent of nutrient addition within alpine meadow systems.

### Effects of Fertilization and Shade on Plant Community Composition

Species composition of the community in each treatment plot changed sequentially during the experimental period (2007–2012) (Figure 2). The reason may be that it takes time to shift community composition because some species gradually disappeared with fertilization and shade, while others gradually increased. For example, *Gramineae* species, such as *P. poophagorum* and *K. cristata*, rapidly increased and dominated in the fertilization plots and shade plots instead of the former dominant species (*T. ruthenica* and *K. capillifolia*) of the initial communities (Supplementary Table 2). *Fabaceae* and *Cyperaceae* species that inhabited in the understory of the community were very sensitive to light limitation and extincted in less than 3 years within the treatment plots (Figures 1G,H and Table 2).

Nonmetric multidimensional scaling ordination verified the low similarity of community composition between the control and the other treatment plots (Figure 2). One possible reason is that an average of 28 species was found in the control plots compared with 21 species in the fertilization plots and 19 species in the shade plots during the experimental period (Table 2). Moreover, the dominant species between the control and the other treatment plots were also different. *T. ruthenica* (8.22%) dominated the control community but absent from fertilization plots and only covered 0.53% in the shade plots (Supplementary Table 2). There was a high similarity between the fertilization and the shade communities for the reason that some species such as *P. poophagorum* and *Anemone obtusiloba* species gradually became more dominant and some species such as *Fabaceae and Cyperaceae* gradually decreased in abundance both in fertilization plots and shade plots. Although fertilization and shade performed a similar negative effect on species diversity, there was no overlap between the F+S and fertilization or shade plots since only one-third of species were found to be survived in the F+S plots (Table 2).

Studies on the relationships between species diversity and ecosystem functioning often showed a unimodal pattern along the primary productivity gradient (O’Connor et al., 2017; Kimmel et al., 2020; Yue et al., 2020). In our experiment, species diversity reduced significantly regardless of aboveground biomass increase due to fertilization or decrease due to shade (Figures 1A,B,D). Interestingly, fertilization increased aboveground biomass with a neutral effect on belowground biomass, resulting in a significant decrease of the root:shoot ratio (Figure 1D and Table 2). The result implied that the coexisting plant species switched the strategy of biomass allocation tending to aboveground organs to promote vegetative growth in response to fertilization, especially for the species with rapid feedback on soil nutrients and light availability fluctuations (e.g., *Gramineae* species). In contrast, artificial shade decreased aboveground and belowground biomass synchronously, resulting in a neutral —change in the root:shoot ratio (Figure 1D and Table 2). The result suggested that the coexist plant species did not adjust the strategy of biomass allocation in response to light limitation due to shade treatment. Therefore, the species loss due to light limitation should have different underlying mechanisms between the fertilization and shade communities.

Previous studies have demonstrated that species loss due to long-term exposure to high nutrient availability was nonrandom (Zhou et al., 2021, 2022) and that the initial biomass increase trend after fertilizer application gradually diminished over time (Isbell et al., 2013; Kimmel et al., 2020; Zhao et al., 2021). Understory species might be severely constrained by light limitation and thus suffered a higher risk of localized exclusion compared with the canopy species (Dickson and Gross, 2013; DeMalach et al., 2017). Our results confirmed that most non-*Gramineae* species, which usually inhabited the understory, were very sensitive to light limitation (Figures 1G,H). Both fertilization and shade dramatically and disproportionally affected the SRA of understory species (Figures 1G,H). In contrast, fast-growing, nutrient-preferring, and taller *Gramineae* species dominated the canopy of the treated communities, contributing to the majority of the community biomass and competitively excluding slow-growing, nutrient-conservative species (Li et al., 2011, 2017; Liu et al., 2015). Furthermore, there were significant interaction effects between fertilization and shade on species diversity and species abundance, and the interaction effects enhanced along the temporal scale (Figure 1 and Table 2), indicating that fertilization and shade performed roughly equal effects on plant communities.

### Effects of Fertilization and Shade on Functional Traits and Their Relationships With Species Relative Abundance

Species traits are the results of functional trade-offs between different plant functions and from adaptive and plastic responses to its biotic and abiotic environments (Dickson et al., 2014; Li et al., 2021). Consistent with Clark et al. (2010) and Dickson et al. (2014), species loss under fertilization and shade in our study could be predicted by plant height and photosynthetic capacity traits, respectively (Figures 3C,D). For example, fertilization facilitated the performance of taller *Gramineae* species but suppressed non-*Gramineae* species in accordance with the previous study that *Gramineae* species had asymmetric access to the higher nitrogen supply and then eliminate non-*Gramineae* species through light competition due to their higher growth rate (Figures 1E,F; Dickson and Gross, 2013; Dickson et al., 2014). Moreover, our results were in contrast to those from a meta-analysis of 37 studies by Suding et al. (2005), which recorded no relationship between species height and species loss. The negative effect of fertilization on non-*Gramineae* species could be attributed to their shorter, rosette life form. In addition, our results clearly showed that there was a strong positive correlation between species height and their SRA in the fertilized plots, with height being a strong predictor of species loss in these plots (Figure 3D). However, this relationship was not observed within communities in either the shade or control plots. This disparity suggested that enhanced plant vegetative growth due to fertilization was an important driver of species loss in the communities of alpine meadows.

Shade promoted species with lower LCP, with an increase in the relative abundance of these species in the shaded plots (Figure 3C). Species with low LCP typically exhibit strong tolerance to light limitation and usually were dominant species in low-light environments (Horn, 1971; Kitao et al., 2016). This pattern was evident in our study system, whereby the SRA of rare species with low LCP (e.g., *Gentiana macrophylla* and *Galium verum*) remarkably increased in shade plots (Supplementary Table 2). We also detected a strong negative correlation between species LCP and their SRA in the artificial shade plots, with LCP being an effective predictor of species loss in these plots (Figure 3C). However, this relationship was not observed within communities in either the fertilization or control plots, indicating that the tolerance capacity of light limitation was an important driver of community assembly for the alpine meadow. Results from the SEMs also supported our theory that both species height and LCP played decisive roles in the process of species loss due to light limitation.

Our previous 3-year field experiment at this study site indicated that fertilization influenced plant species richness by increasing aboveground biomass and livestock can neutralize or mask the negative effects of fertilization on plant species diversity *via* ingesting aboveground biomass (Li et al., 2017). Since competition for light is size asymmetric and increasing aboveground biomass due to fertilization aggravated the light limitation in the understory, we recommend that moderate grazing or mowing should be applied along with the increasing nitrogen deposition or fertilizer application to stabilize the local species diversity in the alpine meadow of the Tibetan Plateau (Sun et al., 2021a,b).

Overall, this study illustrates how artificial shade and fertilization influenced species diversity and community structure and verified the light competition theory as the main drivers of community assembly in the alpine meadow of the Tibetan Plateau. While the responses of community composition to fertilization and shade differed among experimental treatments, traits associated with light capture and shade tolerance determined the pattern of species coexistence in light limitation habitat. Furthermore, our results highlighted that species height and LCP are good indicators for predicting species loss and suggested that species diversity reduction due to fertilization may be minimized by reducing the individual height of canopy species in the alpine meadow (e.g., mowing or grazing).

## Data Availability Statement

The original contributions presented in the study are included in the article/Supplementary Material, further inquiries can be directed to the corresponding authors.

## Author Contributions

JL and ZY contributed to the conception and design of the study. JL organized the database and wrote the first draft of the manuscript. JL, ZY, and LC performed the statistical analysis and wrote the draft of the manuscript. GD and SF provided the funding and revised the manuscript. All authors contributed to manuscript revision, read, and approved the submitted version.

## Conflict of Interest

The authors declare that the research was conducted in the absence of any commercial or financial relationships that could be construed as a potential conflict of interest.

## Publisher’s Note

All claims expressed in this article are solely those of the authors and do not necessarily represent those of their affiliated organizations, or those of the publisher, the editors and the reviewers. Any product that may be evaluated in this article, or claim that may be made by its manufacturer, is not guaranteed or endorsed by the publisher.
