# Supplementary material for: Differential Mechanisms Drive Species Loss Under Artificial Shade and Fertilization in the Alpine Meadow of the Tibetan Plateau
Source: Front Plant Sci. 2022 Feb 8;13:832473. doi: 10.3389/fpls.2022.832473 (PMC8860893; doi:10.3389/fpls.2022.832473)
Supplement: Supplementary file 1 [file Data_Sheet_1.docx]

**Electronic Supplementary Material**

**Table S1** Forty plant species occurs in the alpine meadow of the experimental site. Functional groups consist of the two groups**:** *Gramineae* group and *Non-gramineae* group. Experimental treatments consist of four treatments: CK: control treatment; F: fertilization treatment; S: shade treatment; F+S: synchronous fertilization and shade treatment.

| **Plant species (family name)** | **Family** | **Functional Group** | **Sample plot** |
| --- | --- | --- | --- |
| *Allium beesianum* | *Amaryllidaceae* | Non-gramineae | CK, F, S |
| *Bupleurum malconense* | *Apiaceae* | Non-gramineae |  |
| *Ajania tenuifolia* | *Asteraceae* | Non-gramineae |  |
| *Artemisia sieversiana* | *Asteraceae* | Non-gramineae | CK, S |
| *Anaphalis lacteal* | *Asteraceae* | Non-gramineae | CK, S |
| *Ligularia virgaurea* | *Asteraceae* | Non-gramineae | CK, F, S |
| *Saussurea amara* | *Asteraceae* | Non-gramineae |  |
| *Saussurea chetchozensis* | *Asteraceae* | Non-gramineae | CK, F |
| *Taraxacum mongolicum* | *Asteraceae* | Non-gramineae |  |
| *Carex pseudosupina* | *Cyperaceae* | Non-gramineae | CK, F, S |
| *Kobresia capillifolia* | *Cyperaceae* | Non-gramineae | CK, S |
| *Kobresia humilis* | *Cyperaceae* | Non-gramineae |  |
| *Scirpus pumilus* | *Cyperaceae* | Non-gramineae |  |
| *Euphorbia esula* | *Euphorbiaceae* | Non-gramineae | CK, S |
| *Gentiana macrophylla* | *Gentianaceae* | Non-gramineae | CK, F, S |
| *Halenia elliptica* | *Gentianaceae* | Non-gramineae |  |
| *Geranium calanthum* | *Gentianaceae* | Non-gramineae |  |
| *Trigonella ruthenica* | *Fabaceae* | Non-gramineae | CK, S |
| *Oxytropis kansuensis* | *Fabaceae* | Non-gramineae |  |
| *Tibetia himalaica* | *Fabaceae* | Non-gramineae |  |
| *Pedicularis kansuensis* | *Scrophulariaceae* | Non-gramineae | CK, F, F+S |
| *Veronica eriogyne* | *Plantaginaceae* | Non-gramineae | CK, S |
| *Agrostis gigantea* | *Poaceae* | Gramineae | CK, F |
| *Agrostis trinii* | *Poaceae* | Gramineae | CK, F |
| *Elymus nutans* | *Poaceae* | Gramineae | CK, F, S, F+S |
| *Festuca sinensis* | *Poaceae* | Gramineae |  |
| *Koeleria cristata* | *Poaceae* | Gramineae | CK, F, S, F+S |
| *Leymus secalinus* | *Poaceae* | Gramineae |  |
| *Poa poophagorum* | *Poaceae* | Gramineae | CK, F, S, F+S |
| *Stipa aliena* | *Poaceae* | Gramineae | CK, S |
| *Anemone obtusiloba* | *Ranunculaceae* | Non-gramineae | CK, F, S, F+S |
| *Anemone rivularis* | *Ranunculaceae* | Non-gramineae | CK, F, S, F+S |
| *Delphinium kamaonense* | *Ranunculaceae* | Non-gramineae |  |
| *Ranunculus membranaceus* | *Ranunculaceae* | Non-gramineae |  |
| *Ranunculus tanguticus* | *Ranunculaceae* | Non-gramineae |  |
| *Potentilla anserine* | *Rosaceae* | Non-gramineae |  |
| *Potentilla bifurca* | *Rosaceae* | Non-gramineae |  |
| *Potentilla fragarioides* | *Rosaceae* | Non-gramineae |  |
| *Galium verum* | *Rubiaceae* | Non-gramineae | CK, F, S, F+S |
| *Viola pseudo-bambusetorum* | *Violaceae* | Non-gramineae |  |

**Table S2** Means of species height (SH) and species relative abundance (SRA) among the four treatments: fertilizer addition and ambient light (F), no fertilizer addition and shade (S), synchronous fertilizer addition and shade (F+S), and no fertilizer addition and ambient light (CK) treatment plots in 2012. Species were classified by two functional groups (*Gramineae* group (GG) and *non-Gramineae* group (NGG)). Values with significant difference between the control and treatments plots were in bold (P>0.05). “—” mean that the species had almost eliminated and not sampled from the experiment plots.

| Sampled species | Group | Species height (cm) | | | | Light compensation light | | | | Species relative abundance (%) | | | |
| --- | --- | --- | --- | --- | --- | --- | --- | --- | --- | --- | --- | --- | --- |
|  |  | CK | F | S | F+S | CK | F | S | F+S | CK | F | S | F+S |
| *Stipa aliena* | GG | 15.53 | — | **36.00** | — | 34.42 | — | **24.48** | — | 3.46 | — | **6.32** | — |
| *Agrostis trinii* | GG | 24.33 | **42.01** | — | — | 103.66 | 127.72 | — | — | 0.46 | **8.17** | — | — |
| *Poa poophagorum* | GG | 29.51 | **48.92** | **47.08** | **53.67** | 21.14 | 26.04 | **15.12** | 18.90 | 3.65 | **21.15** | 8.11 | **30.93** |
| *Agrostis gigantea* | GG | 34.03 | **40.06** | — | — | 153.37 | **189.72** | — | — | 3.37 | **11.41** | — | — |
| *Elymus nutans* | GG | 35.04 | **52.42** | **64.50** | **59.75** | 103.77 | **127.72** | **74.16** | 92.71 | 5.48 | 7.17 | 3.48 | 5.02 |
| *Koeleria cristata* | GG | 40.65 | **51.48** | **51.29** | **48.17** | 41.22 | **50.84** | **29.52** | 36.91 | 0.46 | **10.24** | **4.88** | 1.74 |
| *Carex pseudosupina* | NGG | 16.00 | **29.02** | **26.50** | — | 10.08 | 12.41 | 7.20 | — | 3.45 | 3.21 | **10.33** | — |
| *Kobresia capillifolia* | NGG | 28.19 | — | **35.25** | — | 119.91 | — | **85.68** | — | 3.81 | — | **0.73** | — |
| *Trigonella ruthenica* | NGG | 7.59 | — | **14.67** | — | 59.42 | — | **42.48** | — | 8.22 | — | **0.53** | — |
| *Anemone obtusiloba* | NGG | 4.84 | **14.17** | **13.83** | **13.02** | 5.23 | 6.20 | 3.6 | 4.52 | 3.05 | **7.09** | **11.19** | **10.12** |
| *Anaphalis lactea* | NGG | 5.20 | — | 5.34 | — | 84.11 | — | **60.48** | — | 0.91 | — | 0.85 | — |
| *Pedicularis kansuensis* | NGG | 5.53 | 5.00 | — | 5.04 | 69.08 | 85.56 | — | 62.10 | 0.31 | 0.17 | — | 0.17 |
| *Artemisia sieversiana* | NGG | 7.93 | — | **20.33** | — | 82.14 | — | **59.04** | — | 3.65 | — | 1.03 | — |
| *Saussurea chetchozensis* | NGG | 8.85 | 10.67 | — | — | 31.32 | 38.44 | — | — | 4.46 | 4.59 | — | — |
| *Gentiana macrophylla* | NGG | 9.5 | 5.50 | 12.50 | **21.53** | 19.13 | 23.56 | 13.68 | 19.81 | 3.43 | **0.91** | **7.20** | **18.02** |
| *Galium verum* | NGG | 10.13 | **19.33** | **20.27** | — | 22.05 | 27.28 | 15.84 | — | 6.39 | 6.49 | **12.82** | — |
| *Veronica eriogyne* | NGG | 10.25 | — | **22.33** | — | 76.11 | — | **54.72** | — | 4.57 | — | **0.38** | — |
| *Euphorbia esula* | NGG | 10.68 | — | 9.78 | — | 99.53 | — | **71.28** | — | 2.04 | — | 4.22 | — |
| *Anemone rivularis* | NGG | 17.41 | **37.83** | 25.42 | **37.42** | 77.61 | **95.48** | **55.44** | 69.33 | 2.28 | **10.18** | 6.13 | **8.76** |
| *Allium beesianum* | NGG | 20.03 | 5.01 | **31.29** | — | 40.04 | 49.60 | 28.80 | — | 1.83 | 0.5 | 4.56 | — |
| *Ligularia virgaurea* | NGG | 22.67 | 15.00 | 22.70 | — | 132.68 | **163.68** | **95.04** | — | 3.02 | 4.21 | 0.52 | — |

**Fig. S1** Mean monthly temperature and precipitation in the study site over the experimental period (2008/01 to 2012/12).

**

**

**Fig. S2** Schematic representation of the experiment design. Experiment contains 32 subplots (2 m×2 m) , averagely arranged both inside and outside the shade shed (25 m×30 m), and at least 3 m away from the shed edge. There was a 2-m buffer zone between subplots both inside and outside the shade shed. CK: no fertilizer addition and ambient light; F: fertilizer addition and ambient light; S: no fertilizer addition and shade; F+S: synchronous fertilizer addition and shade.

**
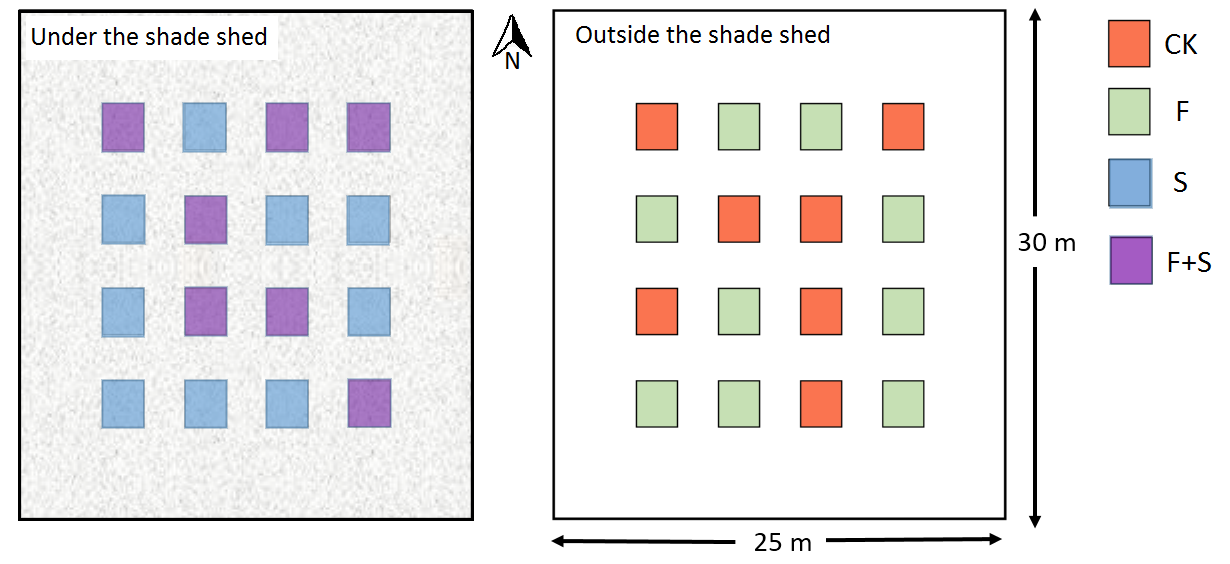
**

**Fig. S3** Principal component analysis (PCA) of matrices of light intensity (including light intensities above plant level and at the heights of 0, 10 and 40 cm above soil surface). The points represent the means ± SE (n = 8) of PC1 and PC2 scores of each treatment (CK: no fertilizer addition and ambient light; F: fertilizer addition and ambient light; S: no fertilizer addition and shade; S+F: synchronous fertilizer addition and shade.). The loadings of PC1 reflect the light intensities at four layers. The PC1 scores of light increase with increasing light.

**

**
